# Florence Chatbot Evaluations, WHO Dialogic Commitment, and Vaccination Intention: The Conditional Role of Misinformation Involvement

**DOI:** 10.3390/healthcare14213671

**Published:** 2026-10-28

**Authors:** Duo Lan, Yicheng Zhu, Yang Cheng

**Affiliations:** 1 School of Digital Media and Design Arts, Beijing University of Posts and Telecommunications, Beijing 100876, China; landuo@bupt.edu.cn; 2 School of Journalism and Communication, Beijing Normal University, Beijing 100875, China; 3 Department of Communication, North Carolina State University, Raleigh, NC 27695, USA

**Keywords:** vaccine chatbot, vaccination intention, involvement, risk perception, dialogic communication, artificial intelligence

## Abstract

**Highlights:**

**What are the main findings?**

- Chatbot Competency and Emotional Connection were positively associated with WHO Dialogic Commitment, which was positively associated with Vaccination Intention.
- Chatbot-Use Risk Perception was negatively associated with WHO Dialogic Commitment at low and average Involvement, but the association was not detectable at high Involvement.

**What are the implications of the main findings?**

- Competent, emotionally responsive communication and transparent privacy practices are design considerations for future chatbot trials, not interventions tested here.
- Misinformation Involvement may shape whether chatbot-channel concerns become negative relational judgments about WHO; the association needs prospective testing.

**Abstract:**

Background/Objectives: Public-health chatbots convey information while representing health organizations. We examined whether evaluations of WHO’s (World Health Organization) Florence chatbot were associated with WHO Dialogic Commitment and Vaccination Intention, and whether misinformation Involvement conditioned the indirect association of Chatbot-Use Risk Perception with Vaccination Intention. Methods: We analyzed a cross-sectional survey of 416 complete-case U.S. respondents with prior chatbot experience who interacted with Florence. Confirmatory factor analysis and demographic-adjusted latent-variable structural equation modeling assessed the main associations. A standardized-composite moderated-mediation model used 5000 bootstrap samples. Latent product-indicator, vaccination-history-adjusted, and full-information maximum-likelihood models provided sensitivity checks. Results: The six-factor measurement model fit well, χ^2^(155) = 316.25, CFI = 0.973, RMSEA = 0.050, SRMR = 0.051. Chatbot Competency and Emotional Connection were positively associated with WHO Dialogic Commitment, which was positively associated with Vaccination Intention. The average Chatbot-Use Risk Perception path was nonsignificant. Its interaction with Involvement was positive (b = 0.079, 95% bootstrap CI [0.023, 0.133]); the indirect association through WHO Dialogic Commitment was negative at low and mean Involvement but not detectable at high Involvement. A latent-interaction sensitivity model yielded a similarly positive interaction. Conclusions: Respondents’ Florence evaluations covaried with perceived WHO dialogue and vaccination-related intentions. Involvement attenuated the negative risk–dialogue association. Mixed initial-vaccination and booster meanings, nonprobability recruitment, and same-session self-report limit causal and population-level interpretations.

## 1. Introduction

### 1.1. Public-Health Chatbots as Organizational Communication Interfaces

Conversational artificial intelligence (AI) is increasingly used for vaccine questions, misinformation correction, and tailored health guidance. Digital-health innovation also includes intelligent systems for mpox monitoring and treatment [1], illustrating that communication chatbots occupy one part of a wider technological response. Reviews suggest potential benefits for vaccine knowledge, attitudes, and intentions, but intervention effects vary by design and follow-up [2,3]. A randomized field trial in Argentina found that a two-way chatbot increased COVID-19 (coronavirus disease 2019) vaccination relative to a one-way reminder [4]. A separate randomized trial of HPV-vaccine messaging found short-term intention gains from a large-language-model chatbot, but not a durable advantage over public-health materials [5]. Analysis of vaccine-chatbot messages shows that users bring diverse vaccine-safety and recommendation concerns to these interfaces [6]. Yet it remains less clear whether an encounter with a health organization’s chatbot is associated with judgments about the organization’s willingness to listen and respond—and whether those judgments relate to vaccination intention.

Health communication is interpreted through users’ prior beliefs, information needs, and the perceived relevance of an issue. Vaccine-chatbot reviews and conversation data show that users bring diverse concerns to these interfaces [2,3,6]. Thus, intentions may reflect not only information exposure but also how a communication encounter is interpreted.

Audiences also interpret AI systems in relation to the roles they appear to perform. Interface cues can shape expectations of a healthcare chatbot’s competence and appropriateness [7]. In a separate AI fact-checking experiment, role-based machine cues and conspiratorial orientation jointly shaped users’ corrective-action intentions [8]. Although that study did not examine health chatbots, it supports considering how prior orientations condition responses to AI-mediated correction. In a vaccine setting, users may judge whether an agent seems responsive to their questions and suitable for organizational health guidance. Perceptions of a specific AI agent and its institutional role may therefore matter for public-health communication.

Florence was presented to respondents as a WHO digital health worker, not as an unbranded AI tool. WHO describes Florence as an interactive channel that can provide information about COVID-19 vaccines and other health topics [9]. In this survey, respondents received the WHO/Florence introduction, interacted with Florence for at least five minutes while addressing three standardized vaccine questions, and then rated both the chatbot and WHO. This sequence permits an examination of cross-target associations: whether a WHO-branded interface is evaluated alongside WHO’s perceived dialogic commitment. It does not establish that the chatbot changed that perception. Prior analyses of the Florence survey program focused on satisfaction, trust, engagement, relationship quality, or organizational support [10,11]. The present study instead focuses on WHO Dialogic Commitment as a relational construct, Vaccination Intention as the outcome, and misinformation Involvement as a condition on the chatbot-risk association.

The conceptual problem is important because users may assess a chatbot’s competence, warmth, and safety as signals of how its sponsoring institution communicates. A systematic review of trust in health organizations identifies competence and transparency as recurring determinants, while cautioning that their meaning varies by setting [12]. The constructs here refer to distinct targets: Chatbot Competency, Emotional Connection, and Chatbot-Use Risk Perception concern Florence; WHO Dialogic Commitment concerns WHO. We test associations across those targets, not their equivalence.

### 1.2. From Chatbot Experience to WHO Dialogic Commitment

Chatbot Competency reflects whether Florence is perceived as competent, knowledgeable, and sensible. Design cues can shape perceived expertise and trust in healthcare chatbots [7], and vaccine-chatbot reviews emphasize credible, accessible responses [3]. In an experiment, chatbot interactivity and empathy were associated with misinformation correction and vaccination intentions, indicating that response qualities can matter beyond simple message delivery [13]. The present construct addresses perceived competence, not objective answer accuracy.

For an organizational chatbot, perceived competency may also signal that the sponsoring organization has invested in accessible and responsive communication. Florence-specific research links functional value to chatbot trust and favorable chatbot experiences to perceived relationship quality with WHO [10,11]. We therefore expect Chatbot Competency to be positively associated with WHO Dialogic Commitment.

**Hypothesis** **H1.** *Chatbot Competency is positively associated with WHO Dialogic Commitment.*

Emotional Connection reflects whether Florence seems caring and understanding. An experimental vaccine-chatbot study found that interactive and empathetic responses influenced correction and vaccination intentions [13]. In a distinct virtual-reality health-communication setting, social presence and emotional responses were associated with self-protective intentions [14]. This adjacent evidence motivates examining affective experience without treating Florence as a person or assuming that warmth alone changes behavior.

Perceived care and understanding may signal organizational attentiveness, qualities central to dialogic communication. In the Florence context, Emotional Connection was positively associated with chatbot trust, and positive chatbot experiences were associated with engagement and perceived relationship quality with WHO [10,11]. Users who experience Florence as caring and understanding may consequently view WHO as more attentive and open to public perspectives. This is a relational attribution, not a claim that the chatbot experiences human emotion.

**Hypothesis** **H2.** *Emotional Connection is positively associated with WHO Dialogic Commitment.*

Chatbot-Use Risk Perception concerns perceived risk in using Florence, particularly privacy and security when disclosing personal information. It does not measure vaccine side-effect risk, disease risk, or misinformation risk. Privacy and security are distinct features of conversational-AI evaluation [15], and privacy risk was negatively associated with chatbot satisfaction in prior Florence research [10]. Health-organization trust research also suggests that transparency and perceived competence matter to institutional evaluations [12].

Concerns about an organizational chatbot’s handling of personal information could accompany weaker perceptions that its sponsoring organization supports safe and responsive dialogue. The cross-target transfer is an empirical question rather than a presumed causal mechanism.

**Hypothesis** **H3.** *Chatbot-Use Risk Perception is negatively associated with WHO Dialogic Commitment.*

### 1.3. WHO Dialogic Commitment and Vaccination Intention

Dialogic communication treats publics as communication partners rather than passive recipients. The organization–public dialogic communication scale emphasizes mutuality, listening, common ground, understanding, and empathy [16]. WHO Dialogic Commitment therefore captures a specific perception of WHO’s willingness to engage responsively, rather than general organizational favorability.

Vaccine acceptance is not solely an information-deficit problem. Biomedical vaccine-safety evidence can also be population-specific, as in a prospective cohort of couples undergoing assisted reproduction [17], whereas general vaccination-intention items compress heterogeneous decisions. Vaccine-chatbot evidence points to credibility, natural interaction, and trust as relevant conditions, while evidence for sustained behavior remains limited [2,3]. The large-scale Vaccine Information Resource Assistant (VIRA) analysis likewise illustrates that users seek answers to heterogeneous vaccine concerns through two-way interfaces [6]. Florence evaluations may therefore be associated not only with information judgments but also with whether respondents see WHO as a responsive communication partner.

Evidence from vaccine-chatbot reviews and intervention research suggests that user experiences and response qualities can relate to vaccine intentions [2,3,13]. Although these studies do not test the institutional-dialogue pathway, they support examining whether perceived WHO Dialogic Commitment is associated with Vaccination Intention.

**Hypothesis** **H4.** *WHO Dialogic Commitment is positively associated with Vaccination Intention.*

### 1.4. Involvement as a STOPS-Inspired Condition on the Chatbot-Risk-to-Dialogue Association

The Situational Theory of Problem Solving (STOPS) proposes that problem recognition, involvement recognition, and constraints shape communicative motivation and action [18]. Involvement here concerns the personal relevance of COVID-19 misinformation, while Chatbot-Use Risk Perception concerns Florence’s channel and information security. We use STOPS to motivate one moderation hypothesis; we do not estimate its full situational-motivation model or recast channel risk as a STOPS construct.

#### 1.4.1. Why Privacy Risk Can Discourage Engagement, but Need Not End It

Privacy and security concerns may deter engagement with digital health channels [19,20]. At the same time, willingness to disclose information can depend on perceived benefit, issue relevance, and relational identification [19,21]. This research does not directly test the Florence interaction. It establishes why a channel-related concern may be weighed against a personally important information need, rather than operating with an identical force for every respondent.

Risk-information models likewise allow for more than one response to concern. The risk-information-seeking-and-processing model proposes that hazard perceptions, affect, information sufficiency, capacity, and channel beliefs shape information seeking and processing [22]. Risk may prompt avoidance or create an information gap that motivates further seeking. Experimental evidence links post-message information seeking to subsequent health-behavior appraisals [23], while the Chatbot-Use Risk Perception Attitude framework predicts more seeking when high threat is accompanied by efficacy rather than helplessness [24].

These perspectives do not establish how highly involved Florence users respond to privacy concerns, but they indicate that concern need not produce uniform disengagement. Responses depend partly on what is at stake and whether users remain motivated to process information. WHO Dialogic Commitment captures the relevant relational judgment—whether WHO appears attentive and responsive—rather than disclosure or technology adoption itself.

#### 1.4.2. Involvement as Personal Relevance and Motivation to Process

STOPS treats personally relevant problems as stronger prompts for communicative action [18]. Involvement recognition has been associated with cancer-information seeking and online access [25], and with COVID-19 information acquisition in an extended STOPS study [26]. The risk-information-seeking-and-processing account likewise allows perceived information need and channel beliefs to shape continued seeking [22]. These perspectives motivate, but do not prove, the proposed privacy-risk attenuation in this specific chatbot setting.

The present survey did not measure the full STOPS motivational sequence. Accordingly, this study takes a narrower approach: it examines whether the personal relevance of misinformation changes how concern about the chatbot channel relates to perceptions of WHO.

Involvement can alter responses to health-risk information rather than exerting a uniformly positive effect. In an experiment on alcohol risks, statistical evidence influenced highly involved participants, whereas exemplars were more influential among less involved participants [27]. This finding supports treating involvement as a boundary condition, although it does not by itself predict attenuation of privacy-risk effects.

#### 1.4.3. A Cross-Target Attenuation Account

When misinformation has low personal relevance, privacy or security concerns about Florence may provide a sufficient reason to disengage. With little incentive to tolerate uncertainty or seek clarification, users may more readily generalize channel-related risk to a negative assessment of WHO’s dialogic commitment.

When misinformation is personally relevant, users may have stronger reasons to seek information from WHO even if they have reservations about Florence. STOPS and risk-information models connect personal relevance, information sufficiency, and channel evaluation with communicative action [18,22,25]. Under this account, the same degree of channel concern would be less strongly associated with a negative judgment of WHO’s dialogic commitment among highly involved respondents. That is a cross-target attenuation hypothesis, not a claim that involvement removes privacy risk.

The opposite pattern is also plausible: highly involved users might scrutinize privacy cues more intensely and become especially critical of a risky channel. Experimental evidence that outcome-relevant involvement changes responses to health-risk information underscores why the direction must be tested rather than assumed [27]. The hypothesized attenuation rests specifically on the mismatch between the personally relevant misinformation problem and the perceived risk of the communication channel.

Research on healthcare-chatbot design indicates that interface cues can shape expertise and trust judgments [7], while vaccine-chatbot evidence shows variation in user concerns and response qualities [6,13]. Although these studies do not test Florence, they support the expectation that prior orientations affect the interpretation of AI-mediated communication.

The model uses STOPS selectively. Involvement is not assumed to moderate every risk effect, and chatbot-use risk is not treated as a STOPS construct. The hypothesis is limited to a channel-related risk evaluated in the context of a personally relevant misinformation problem.

**Hypothesis** **H5.** *Involvement moderates the association between Chatbot-Use Risk Perception and WHO Dialogic Commitment, such that the negative association is weaker at higher levels of Involvement.*

### 1.5. Conditional Indirect Association with Vaccination Intention

These arguments imply a first-stage moderated-mediation model. Chatbot-Use Risk Perception is associated with WHO Dialogic Commitment, which is associated with Vaccination Intention, while Involvement conditions the first association. Chatbot Competency and Emotional Connection remain predictors of WHO Dialogic Commitment in the model.

At lower Involvement, privacy or security concerns should be more strongly associated with lower WHO Dialogic Commitment and, indirectly, lower Vaccination Intention. This indirect association should weaken as Involvement increases. The hypothesis concerns this specific cross-target pattern and does not assume that involvement neutralizes privacy risk in general.

The model links evaluations of the chatbot channel, perceptions of the sponsoring organization, and vaccination intention. Because the data are cross-sectional, the hypotheses concern conditional associations rather than causal effects.

**Hypothesis** **H6.** *Involvement conditions the indirect association of Chatbot-Use Risk Perception with Vaccination Intention through WHO Dialogic Commitment, such that the indirect association is more negative at lower levels of Involvement and attenuates as Involvement increases.*

**Figure 1:**
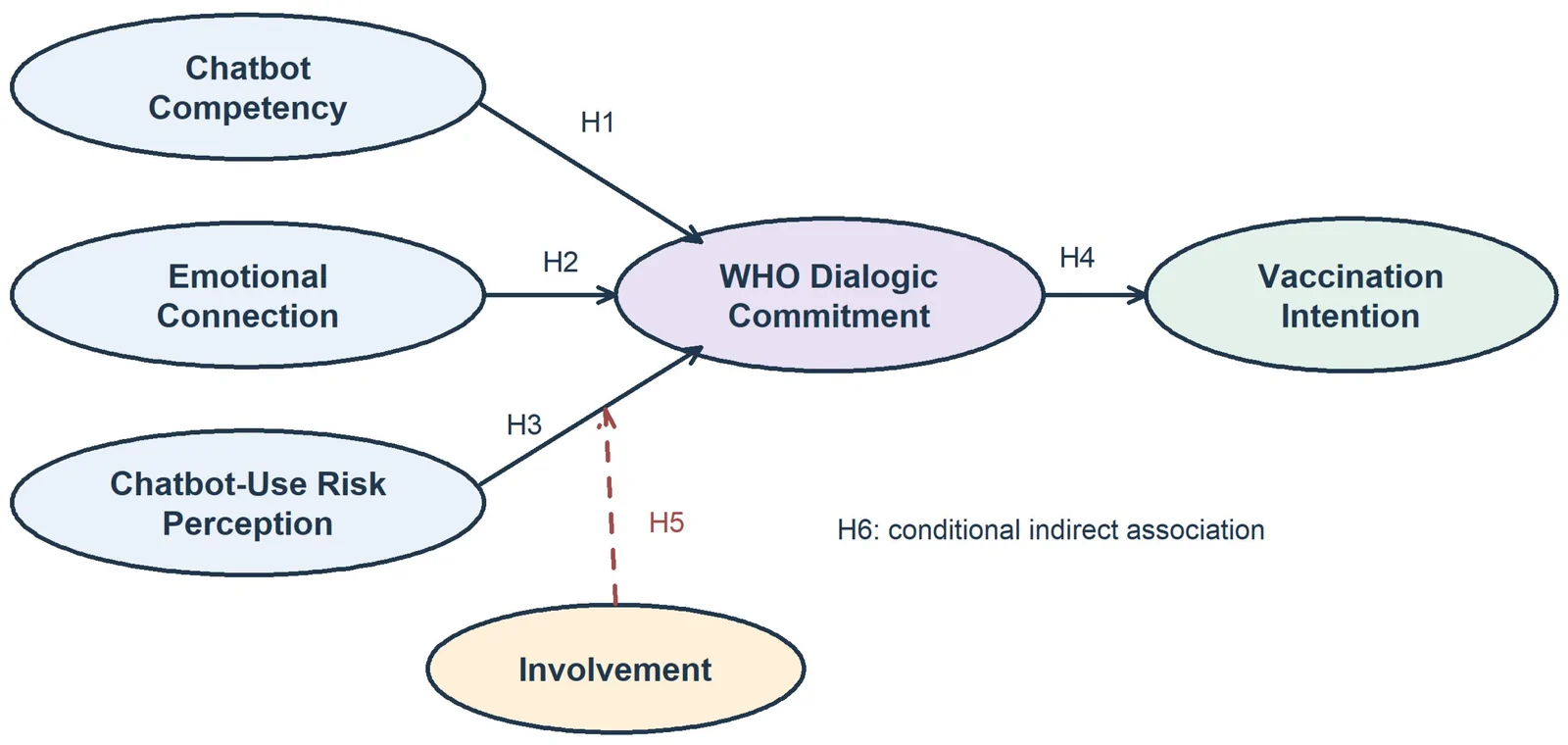
Hypothesized conditional association model with latent constructs. Note. Ovals represent hypothesized latent constructs; item indicators and covariates are omitted for readability. The dashed arrow marks moderation of the Chatbot-Use Risk Perception–WHO Dialogic Commitment association. H6 concerns the resulting conditional indirect association with Vaccination Intention.

The hypothesized conditional process model is shown in Figure 1.

## 2. Materials and Methods

### 2.1. Study Design, Participants, and Procedure

This cross-sectional survey was fielded by Dynata in early 2023. An earlier Florence report describes census-aligned quota sampling; the present secondary analysis has no independent quota-calibration or sampling-frame documentation [11]. Eligibility required U.S. residence and prior chatbot use, so the relevant population is U.S. chatbot-experienced adults reachable through Dynata’s panel, not all U.S. adults. Respondents received an introduction to WHO’s vaccine-misinformation campaign and Florence, a WHO-branded interactive digital health worker [9]. They were instructed to interact with Florence for at least five minutes while addressing three standardized COVID-19 vaccine questions before rating the chatbot, WHO, and their vaccination-related intentions. The archived file records the survey responses, not a time-stamped transcript of each interaction or objectively verified exposure duration.

Of 3595 exported records, 3519 contained a completed eligibility screener. The prior-chatbot-use filter retained 1993 respondents who reported using chatbots at least sometimes; respondents selecting “almost never” did not proceed. Three subsequent attention checks—two on Florence’s vaccine information and one identifying WHO as the responsible United Nations agency—left 455 early-check-qualified cases. A later end-of-survey check was not enforced because it was not necessary for entry into the focal item blocks and would have imposed an additional post hoc exclusion. The present complete-case analysis retained 416 of the 455 cases (91.4%) with all focal indicators and prespecified demographic covariates observed. The flow reflects screening and survey branching as well as missing responses; it should not be presented as 89% attrition from an initially eligible analytic sample.

The 39 excluded eligible respondents had item- or block-level missingness. Included and excluded eligible respondents differed in education, income, and race in the prior audit, so complete-case estimates may be selection-sensitive. We therefore compared the focal SEM (Structural Equation Modelling) paths with a full-information maximum-likelihood (FIML) analysis of all 455 eligible cases under a missing-at-random assumption. FIML is a sensitivity analysis, not proof that missingness is ignorable; no multiple imputation or probability weighting was applied. Institutional review board approval was granted by North Carolina State University (Protocol No. 25489).

The complete-case sample included 67 respondents (16.1%) reporting no previous COVID-19 vaccination, 125 (30.0%) vaccinated within the preceding 12 months, and 224 (53.8%) vaccinated more than a year earlier. Because every Vaccination Intention item says “COVID-19 vaccine or a booster dose,” the same score can mean initial vaccination for one respondent and a booster for another. We retained the survey’s original wording and checked whether adjusting for vaccination status changed the main latent paths; an exploratory status-by-dialogue interaction tested whether the dialogue–intention association differed detectably between ever- and never-vaccinated respondents. Neither analysis establishes measurement equivalence of the outcome across groups.

### 2.2. Measures

Focal items used 5-point agreement scales (1 = strongly disagree, 5 = strongly agree). Appendix A provides the wording administered to respondents and identifies the measurement sources from which the items were adapted. Cronbach’s α, composite reliability (CR), average variance extracted (AVE), standardized CFA (Confirmatory Factor Analysis) loadings, and heterotrait–monotrait (HTMT) ratios are reported below. Means of the items formed the standardized composites used for the conditional-process analysis.

#### 2.2.1. Chatbot Competency

Chatbot Competency was measured with three items evaluating Florence as competent, knowledgeable, and sensible (α = 0.873). The items were adapted from selected descriptors in the Perceived Intelligence dimension of the Godspeed Questionnaire Series [28] and reformulated as Likert-type statements for the Florence chatbot context. Cheng and Wang [11] provide the corresponding application of these items in the Florence context.

#### 2.2.2. Emotional Connection

Emotional Connection was assessed with three items indicating that Florence cares about the respondent, elicits an emotional connection, and seems to understand the respondent (α = 0.923). The Florence study adapted this measure from Araujo [29].

#### 2.2.3. Chatbot-Use Risk Perception

Chatbot-Use Risk Perception was assessed with three adapted items covering general risk in using Florence and concern that its security systems or privacy protections may not adequately protect personal information (α = 0.796) [30]. The items were adapted from technology-adoption research; they do not assess vaccine adverse events, COVID-19 disease risk, or misinformation exposure.

#### 2.2.4. WHO Dialogic Commitment

WHO Dialogic Commitment was measured with five items assessing whether the WHO effectively handled public opinions, communicated common ground, sought correct understanding, expressed empathy, and attended to what the public said (α = 0.932). The items represent the mutuality/listening dimension of organization–public dialogic communication and were adapted from the organization–public dialogic communication (OPDC) scale of Yang et al. [16].

#### 2.2.5. Vaccination Intention

Vaccination Intention was assessed with three items addressing respondents’ willingness to receive a COVID-19 vaccine or booster dose soon, if making the decision at the present time, and in the future if necessary (α = 0.937). The items were adapted to the COVID-19 vaccination context from Nan and Madden’s [31] measure of intentions to receive HPV vaccination, which was itself adapted from Rothman et al. [32]. The original likelihood questions were reformulated as Likert-type agreement statements.

#### 2.2.6. Involvement

Involvement was assessed with three items concerning the personal relevance and potential consequences of COVID-19 misinformation for respondents or people close to them (α = 0.777). The items operationalize involvement recognition in STOPS [18]. Involvement is examined as a theory-guided moderator; the model is not a full test of STOPS.

#### 2.2.7. Covariates

All primary models adjusted for gender, education, household income, race, and political tendency, selected before model probing because these characteristics may be related to health-information access, institutional evaluation, or vaccination intention. Education and income serve as broad socioeconomic indicators; political tendency captures a potentially salient vaccine-attitude context; race and gender capture demographic heterogeneity rather than biological effects. Male was compared with female; education entered as an ordered category; income used categorical indicators including a “prefer not to answer” category; race was coded White alone versus other reported categories because several cells were sparse; political tendency used Republican and Independent/self-described indicators, with Democrat as reference. Age and previous vaccination status were added in sensitivity analyses because both may influence intention. The archived data do not contain a defensible pre-Florence WHO-trust measure or a pre-exposure vaccination-attitude measure, so these could not be controlled. A WHO satisfaction item collected after the Florence encounter was not treated as baseline trust.

### 2.3. Analytic Strategy

All analysis was performed with R (version 4.5.2). We first estimated a six-factor CFA (including Involvement) and summarized standardized loadings, α, CR, AVE, Fornell–Larcker comparisons [33], and HTMT ratios. A single-factor CFA using the same 20 indicators provided a limited common-method diagnostic; poor one-factor fit cannot rule out all method covariance. We then fitted a demographic-adjusted five-factor latent SEM for H1–H4 (N = 416), with paths from Chatbot Competency, Emotional Connection, and Chatbot-Use Risk Perception to WHO Dialogic Commitment and from WHO Dialogic Commitment to Vaccination Intention. Covariates predicted all latent constructs. Sensitivity models added age and vaccination status or used FIML for all 455 eligible cases.

H5 and H6 were tested with standardized item-mean composites in a first-stage moderated-mediation model on the same 416 cases. Chatbot-Use Risk Perception, Involvement, their product, Chatbot Competency, Emotional Connection, and covariates predicted WHO Dialogic Commitment; Vaccination Intention was regressed on WHO Dialogic Commitment and the same variables. Five thousand nonparametric bootstrap resamples yielded percentile intervals for the interaction, conditional effects at −1 SD, mean, and +1 SD Involvement, conditional indirect associations, and the index of moderated mediation [34]. The composite model provides directly interpretable conditional effects and a Johnson–Neyman plot but does not correct the product term for measurement error. To assess whether the interaction depended on that choice, we separately fitted a product-indicator latent-interaction SEM using three matched, mean-centered risk-by-involvement item products orthogonalized to their constituent indicators. Its interaction coefficient is on a different scale from the composite coefficient; we compare direction and statistical support, not numerical equality. Both models are observational and do not establish mediation in a causal sense.

### 2.4. Reporting and Transparency Notes

Because all focal variables came from one self-report survey session, common-method covariance, reverse causation, and unmeasured pre-existing attitudes remain possible. Appendix A prints the administered item wording; source citations identify the measurement lineage and adaptation context. Appendix B provides the full HTMT matrix and key sensitivity results. We distinguish latent main-effects estimates from composite conditional-process estimates and use associative language throughout.

## 3. Results

### 3.1. Preliminary Analyses and Measurement Quality

Table 1 describes the 416 complete-case respondents. The six scales had acceptable internal consistency (α = 0.777–0.937). Table 2 summarizes the full measurement model; the administered item wording appears in Appendix A.

The six-factor CFA fit well, χ^2^(155) = 316.25, *p* < 0.001, Comparative Fit Index (CFI) = 0.973, Tucker-Lewis Index (TLI) = 0.967, Standardized Root Mean Square Residual (SRMR) = 0.051, Root Mean Square Error of Approximation (RMSEA) = 0.050, 90% confidence interval (CI) [0.042, 0.058], *p* value for the test of close fit (pclose) = 0.488. All standardized loadings were 0.667–0.926; all AVEs exceeded 0.50. The highest HTMT ratio was 0.716, below the conventional 0.85 threshold, providing additional support for discriminant validity without claiming the constructs are perfectly distinct. A single-factor CFA using the same indicators fit much worse, χ^2^(170) = 2626.02, *p* < 0.001, CFI = 0.593, TLI = 0.545, SRMR = 0.126, RMSEA = 0.186, 90% CI [0.180, 0.193]. This comparison makes a single common factor implausible as a complete account of the covariance but does not eliminate shared-method bias. The five-factor CFA for the main SEM also fit well, χ^2^(109) = 210.14, CFI = 0.981, TLI = 0.977, SRMR = 0.046, RMSEA = 0.047.

The demographic-adjusted five-factor SEM fit well, χ^2^(244) = 369.22, *p* < 0.001, CFI = 0.978, TLI = 0.970, SRMR = 0.036, RMSEA = 0.035, 90% CI [0.028, 0.042], pclose = 1.000. Table 3 gives the focal paths. The covariate-adjusted estimates are associations between latent constructs, not effects of a randomized chatbot treatment.

### 3.2. H1: Chatbot Competency and WHO Dialogic Commitment

H1 predicted that Chatbot Competency would be positively associated with WHO Dialogic Commitment. As shown in Table 3, the association was positive, β = 0.463, *p* < 0.001. H1 was supported.

### 3.3. H2: Emotional Connection and WHO Dialogic Commitment

H2 predicted that Emotional Connection would be positively associated with WHO Dialogic Commitment. Emotional Connection was positively associated with WHO Dialogic Commitment, β = 0.379, *p* < 0.001. H2 was supported.

### 3.4. H3: Chatbot-Use Risk Perception and WHO Dialogic Commitment

H3 predicted a negative average association between Chatbot-Use Risk Perception and WHO Dialogic Commitment. The latent SEM coefficient was negative but not statistically significant, β = −0.071, *p* = 0.068 (Table 3). H3 was not supported. This result does not establish a generally negative risk–dialogue association; H5 instead examines whether that association varies by Involvement.

### 3.5. H4: WHO Dialogic Commitment and Vaccination Intention

H4 predicted that WHO Dialogic Commitment would be positively associated with Vaccination Intention. This association was positive and statistically significant, β = 0.553, *p* < 0.001. H4 was supported.

### 3.6. H5: Involvement as a Moderator of the Chatbot-Use Risk Perception–WHO Dialogic Commitment Association

H5 predicted attenuation of the Chatbot-Use Risk Perception–WHO Dialogic Commitment association as Involvement increased. In the adjusted composite model (N = 416), the product term was positive, b = 0.079, 95% bootstrap CI [0.023, 0.133], bootstrap *p* = 0.007. Its incremental R^2^ was 0.009 and partial R^2^ was 0.021. These modest effect-size estimates and the conditional effects in Table 4 indicate attenuation toward zero, not a reversal to a beneficial effect of privacy/security concern. A nonsignificant average path in the latent main-effects model is compatible with heterogeneous conditional associations.

**Figure 2:**
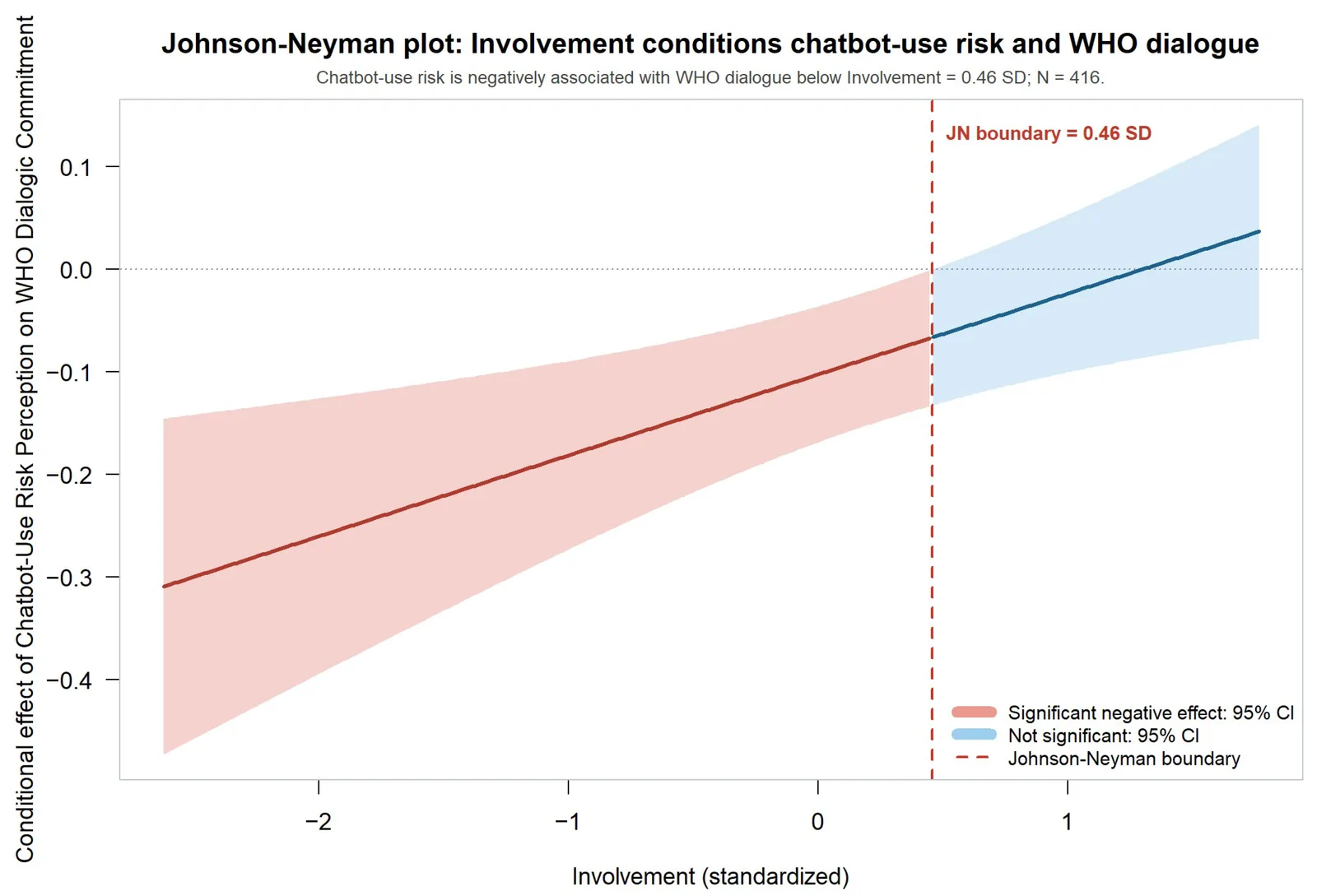
Johnson–Neyman plot of Involvement moderation of the chatbot-use risk–WHO dialogue association. Note. The solid line is the conditional association of Chatbot-Use Risk Perception with WHO Dialogic Commitment across standardized Involvement; bands are 95% confidence intervals, and the dashed line is the Johnson–Neyman boundary. The adjusted composite model uses N = 416. The plot does not indicate a causal effect.

The risk–dialogue association was negative at low and mean Involvement but not distinguishable from zero at high Involvement (Table 4). The Johnson–Neyman region appears in Figure 2. These are within-sample conditional associations; the finding supports H5 without implying a general negative effect across the entire Involvement range.

### 3.7. H6: Conditional Indirect Association of Chatbot-Use Risk Perception with Vaccination Intention

H6 predicted that Involvement would condition the indirect association of Chatbot-Use Risk Perception with Vaccination Intention through WHO Dialogic Commitment. In the composite outcome equation, WHO Dialogic Commitment was positively associated with Vaccination Intention, b = 0.471, *p* < 0.001. The index of moderated mediation was 0.037, 95% bootstrap CI [0.011, 0.067], bootstrap *p* = 0.007. This indicates variation in the modeled indirect association across Involvement, not a proven causal mediation mechanism.

As Table 4 shows, the indirect association was negative at low and mean Involvement, whereas its high-Involvement interval crossed zero. The pattern supports H6 as a conditional indirect association and is consistent with attenuation of the first-stage risk–dialogue link.

Figure 2 shows the Johnson–Neyman region for the conditional Chatbot-Use Risk Perception–WHO Dialogic Commitment association.

### 3.8. Measurement and Model Sensitivity Checks

A product-indicator latent-interaction SEM converged and showed a positive Chatbot-Use Risk Perception × Involvement path to WHO Dialogic Commitment, B = 0.112, SE = 0.036, 95% CI [0.042, 0.182], *p* = 0.002; standardized β = 0.128. This supports the interaction’s direction and statistical detectability when measurement error is modeled, although its coefficient is not directly comparable with the composite b = 0.079. The latent interaction was estimated as a sensitivity model rather than used to replace the bootstrap conditional indirect estimates. Robust fit was χ^2^(401) = 501.30, CFI = 0.983, TLI = 0.978, SRMR = 0.039, RMSEA = 0.026.

Adding age and prior vaccination status to the latent SEM gave standardized paths of 0.445 (Competency → Dialogue), 0.392 (Emotional Connection → Dialogue), −0.058 (Chatbot-Use Risk Perception → Dialogue; *p* = 0.126), and 0.511 (Dialogue → Intention), preserving the focal pattern. The exploratory Dialogue × ever-vaccinated interaction in an adjusted composite outcome model was not detected, b = −0.076, *p* = 0.361; this does not establish equivalent item meaning across vaccination histories. FIML across all 455 early-check-qualified cases likewise gave corresponding paths of 0.469, 0.377, −0.064 (*p* = 0.098), and 0.543 (Appendix B). These checks reduce concern that the main direction is an artifact of the 39 excluded cases, but they do not remove possible nonrandom selection or uncontrolled prior attitudes.

## 4. Discussion

### 4.1. Overview of Findings

Chatbot Competency and Emotional Connection were positively associated with WHO Dialogic Commitment, which was positively associated with Vaccination Intention. The average Chatbot-Use Risk Perception path was not statistically significant. In the conditional-process model, its negative association with WHO Dialogic Commitment was detectable at low and mean Involvement but not at high Involvement; the corresponding indirect association showed the same attenuation. The latent-interaction sensitivity analysis agreed on the interaction’s direction.

These are associations among perceptions measured in one survey session. They are consistent with—but cannot establish—a relational account in which a WHO-branded chatbot is evaluated alongside the institution’s perceived openness to dialogue.

### 4.2. Extending Research on Florence and Public-Health Chatbots

Earlier Florence studies examined chatbot satisfaction, trust, engagement, relationship quality, or organizational support [10,11]. This analysis asks a different theoretical question: whether WHO Dialogic Commitment, a specific judgment about institutional listening and mutual understanding, is associated with Vaccination Intention, and whether misinformation Involvement conditions an indirect association from chatbot-channel risk. The constructs, hypotheses, and conditional-process test differ from those prior reports, though the shared survey limits claims of independent replication.

WHO Dialogic Commitment concerns perceived listening, empathy, common ground, and mutual understanding, rather than broad organization–public relationship quality [16]. Its positive association with Vaccination Intention suggests that relational communication may connect chatbot encounters with vaccine-related intentions.

The finding is compatible with evidence that accessible and responsive chatbots can support vaccine communication, while effect heterogeneity and sustained behavior remain uncertain [2,3,13]. A large field trial found improved COVID-19 vaccine uptake from a two-way chatbot compared with a one-way reminder [4], whereas an HPV-vaccine trial found short-term intention gains without a sustained advantage over public-health materials [5]. These interventions and populations differ from Florence. Observational vaccine-chatbot conversations also contain diverse safety and recommendation questions [6], but none of this evidence shows that interacting with Florence changed the present respondents’ intentions. The organizational-interface interpretation remains a plausible account of cross-target covariance, not a demonstrated transfer effect.

### 4.3. Competency and Emotional Connection as Complementary Chatbot Signals

Chatbot Competency was positively associated with WHO Dialogic Commitment. Users who viewed Florence as competent, knowledgeable, and sensible were also more likely to view WHO as dialogically committed. Consistent with research linking healthcare-chatbot expertise cues to trust [7], perceived competence may signal an institution’s capacity to address public concerns accurately and responsively.

Emotional Connection was independently associated with WHO Dialogic Commitment. Perceived care and understanding may be interpreted as organizational attentiveness rather than solely as a response to a machine. Together, the competency and emotional-connection findings indicate that both cognitive credibility and affective responsiveness contribute to perceptions of organizational dialogue.

### 4.4. Why Chatbot-Use/Privacy–Security Risk Is Conditional on Involvement

H3’s average Chatbot-Use Risk Perception path was negative but not statistically significant. H5 and H6 concern a different claim: within the composite model, the negative risk–dialogue and indirect associations were detectable at low and mean Involvement and attenuated toward zero at high Involvement. The modest interaction effect size and cross-sectional design counsel against describing privacy concern as uniformly harmful or high Involvement as protective in a causal sense.

Chatbot-Use Risk Perception measured concern about using Florence and disclosing personal information—not vaccine adverse events, disease severity, or misinformation exposure. This distinction is central to the theory: the moderator concerns personal relevance of the misinformation problem, whereas the predictor concerns the channel used to address it. Privacy/security and institutional trust may be connected, but the present design cannot determine which evaluation came first [12,15].

A STOPS-inspired interpretation is that personally relevant misinformation may sustain interest in WHO information despite channel concerns, while low-involvement users may have less reason to tolerate uncertainty about the channel [18]. The result is a focused extension of involvement recognition, not a test of STOPS as a whole. The alternative that involvement intensifies attention to privacy failures remains viable in other designs and settings.

### 4.5. Theoretical Contributions

The study connects chatbot-level evaluations with organization-level judgments but does not assume these constructs have a common target. Its contribution is to test their covariance in a WHO-branded setting and to distinguish dialogic commitment from broader relationship quality.

It also places Vaccination Intention in the model. The conditional indirect association through WHO Dialogic Commitment extends beyond chatbot satisfaction or trust, although its statistical form does not establish a temporal or causal sequence.

Finally, the study offers a focused application of STOPS. It specifies how the personal relevance of misinformation may condition the relational implications of privacy and security concerns while preserving the distinct targets of Involvement and Chatbot-Use Risk Perception.

### 4.6. Practical Implications

The findings suggest design questions for future trials rather than confirmed interventions. Researchers could experimentally vary source labeling, answer accuracy, explanation of uncertainty, and human-referral options, then examine whether these features alter perceived competence and WHO dialogue. None of those specific design features was manipulated here.

Similarly, future studies could test whether attentive language or transparent acknowledgment of the limits of automated advice changes Emotional Connection and institutional judgments. The present data show associations with perceived emotional responsiveness, not the effectiveness of a particular conversational script.

Clear statements about data collection, retention, and security may be worth testing—especially among users with low misinformation Involvement—because chatbot-use concerns had a more negative association with perceived WHO dialogue in that subgroup. This is a hypothesis for intervention research, not evidence that a specific privacy notice would improve vaccination intention.

### 4.7. Limitations and Future Research

First, all key variables were collected through one cross-sectional self-report session after the Florence encounter. This design does not establish temporal ordering and permits common-method covariance, consistency motifs, reverse causation, and confounding by prior WHO trust or vaccine attitudes. The six-factor CFA fit far better than a single-factor CFA, but that diagnostic does not rule out shared-method bias. Longitudinal or randomized studies should measure prior attitudes and organizational trust before exposure, assign different chatbot communication strategies, repeat outcomes after exposure, and, where feasible, observe later vaccination behavior.

Second, Dynata quota sampling was not a probability sample of U.S. adults. Eligibility required prior chatbot use and willingness to interact with Florence; health literacy, digital access, institutional trust, and vaccine attitudes may therefore differ from the wider population. Thirty-nine of 455 early-check-qualified respondents were excluded from complete-case estimation, with observed differences in education, income, and race. FIML retained the principal path pattern under its missing-at-random assumption, but it cannot correct unmeasured selection or missing-not-at-random processes. The composite interaction may still contain measurement-error attenuation despite the supportive latent product-indicator sensitivity result.

Third, the vaccine-or-booster wording blends initial-vaccination and booster decisions. Status adjustment and an exploratory status interaction did not alter the principal association pattern, but these checks are not evidence of measurement equivalence, and the never-vaccinated subgroup was small. Prior WHO exposure and baseline vaccination attitudes were not available as defensible pre-encounter measures. Finally, the risk items concern the chatbot channel, and Involvement represents only one element of STOPS. Future studies should separate vaccination decisions and risk domains while testing the full theoretical sequence prospectively.

## 5. Conclusions

In this cross-sectional sample of chatbot-experienced U.S. respondents, Chatbot Competency and Emotional Connection were positively associated with WHO Dialogic Commitment, which was positively associated with Vaccination Intention. H3’s average Chatbot-Use Risk Perception path was not supported. H5 and H6 instead indicate conditional associations: the negative risk–dialogue link and corresponding indirect association were detectable at low and mean Involvement, then attenuated at high Involvement.

These findings motivate further tests of chatbot competence, affective responsiveness, institutional dialogue, and privacy communication. They do not demonstrate that changing Florence or WHO communication would change vaccination behavior; that question requires prospective or experimental evidence.

## Figures and Tables

**Table 1:** Characteristics of the complete-case analytic sample (N = 416).

Characteristic	Category	n (%)
Age	18–29	81 (19.5)
	30–44	136 (32.7)
	45–59	100 (24.0)
	60+	99 (23.8)
Gender	Male	171 (41.1)
	Female	245 (58.9)
Education	High school or less	115 (27.6)
	Some college or associate	92 (22.1)
	Bachelor’s	122 (29.3)
	Graduate	87 (20.9)
Household income	$40,000 or less	136 (32.7)
	$40,001–$80,000	122 (29.3)
	Over $80,000	156 (37.5)
	Prefer not to answer	2 (0.5)
Race	White alone	289 (69.5)
	Black alone	55 (13.2)
	Asian alone	24 (5.8)
	Other or multiracial	48 (11.5)
Political tendency	Democrat	207 (49.8)
	Republican	132 (31.7)
	Independent	70 (16.8)
	Other self-described	7 (1.7)
Prior COVID-19 vaccination	No	67 (16.1)
	Within past 12 months	125 (30.0)
	More than a year ago	224 (53.8)
Prior chatbot use	Most of the time	70 (16.8)
	Always	58 (13.9)
	About half the time	95 (22.8)
	Sometimes	193 (46.4)

Note. Percentages are calculated within the complete-case sample. “Other or multiracial” includes categories too small to report individually. Prior chatbot use was required by the screener; the categories indicate frequency, not experimental assignment.

**Table 2:** Descriptive and measurement-quality summary (N = 416).

Construct	Items	M (SD)	Loading Range	α	CR	AVE
Chatbot Competency	3	3.86 (0.80)	0.807–0.874	0.873	0.875	0.699
Emotional Connection	3	3.01 (1.08)	0.870–0.911	0.923	0.924	0.802
Chatbot-Use Risk Perception	3	3.13 (0.93)	0.693–0.861	0.796	0.804	0.581
WHO Dialogic Commitment	5	3.63 (0.94)	0.835–0.868	0.932	0.932	0.733
Vaccination Intention	3	3.77 (1.16)	0.904–0.926	0.937	0.938	0.834
Involvement	3	3.39 (0.91)	0.667–0.894	0.777	0.796	0.570

Note. Loadings are standardized six-factor CFA estimates. CR = composite reliability; AVE = average variance extracted. All AVEs exceeded 0.50; the maximum pairwise HTMT was 0.716 (full matrix in Appendix B). M = mean; SD = Standard Deviation.

**Table 3:** Focal paths in the demographic-adjusted latent SEM (N = 416).

Hypothesis	Path	B Standard Error (SE)	Standardized β	95% CI for B	*p*	Decision
H1	Chatbot Competency to WHO Dialogic Commitment	0.591 (0.071)	0.463	[0.452, 0.730]	<0.001	Supported
H2	Emotional Connection to WHO Dialogic Commitment	0.357 (0.048)	0.379	[0.263, 0.450]	<0.001	Supported
H3	Chatbot-Use Risk Perception to WHO Dialogic Commitment	−0.082 (0.045)	−0.071	[−0.171, 0.006]	0.068	Not supported
H4	WHO Dialogic Commitment to Vaccination Intention	0.675 (0.058)	0.553	[0.562, 0.788]	<0.001	Supported

Note. B and 95% CIs are unstandardized; β is standardized. Models adjust for gender, education, income, race, and political tendency. H3 tests an average main-effect path and is not supported

**Table 4:** Conditional risk–dialogue and indirect associations by Involvement (N = 416).

Involvement	Risk → WHO b [95% CI]	*p*	Indirect b [95% CI]	*p*
Low (−1 SD)	−0.181 [−0.285, −0.069]	0.002	−0.085 [−0.149, −0.029]	0.002
Mean	−0.102 [−0.168, −0.030]	0.005	−0.048 [−0.088, −0.013]	0.005
High (+1 SD)	−0.023 [−0.086, 0.042]	0.478	−0.011 [−0.041, 0.019]	0.478

Note. Composite scores were standardized before estimation. CIs are percentile bootstrap intervals from 5000 resamples. The same 416 respondents contributed to the latent main-effects SEM and composite conditional-process model.

## Data Availability

The data presented in this study are not publicly available in order to protect participant privacy and confidentiality. De-identified data may be made available from the corresponding authors upon reasonable request.

## Appendix A. Measurement Sources and Administered Item Wording

All measures were administered as 5-point agreement items (1 = strongly disagree, 5 = strongly agree). The entries below reproduce the wording administered in this survey, as recorded in its archived metadata. Items were adapted to the Florence chatbot, WHO, COVID-19 vaccination or boosters, and COVID-19 misinformation context. The citations identify the measurement lineage; the appendix does not claim to reproduce any complete source instrument.

### Appendix A.1. Chatbot Competency

Measurement lineage: Selected perceived-intelligence descriptors from the Godspeed Questionnaire Series [28], applied to Florence in the earlier survey report [11]. Administered items:

Cronbach’s α: 0.873

1. I believe that the chatbot is competent.

2. I believe that the chatbot is knowledgeable.

3. I believe that the chatbot is sensible.

### Appendix A.2. Emotional Connection

Measurement lineage: Adapted for the chatbot context from Araujo [29]. Administered items:

Cronbach’s α: 0.923

1. I feel that this chatbot cares about me.

2. I feel emotionally connected with the chatbot.

3. I feel as though the chatbot really understands me.

### Appendix A.3. Chatbot-Use Risk Perception

Measurement lineage: Technology-use and privacy-risk items adapted from Slade et al. [30]. Administered items:

Cronbach’s α: 0.796

1. My decision to use the chatbot involves a certain level of risk.

2. The security systems built into the chatbot may NOT be strong enough to protect my personal information.

3. I do NOT feel totally safe providing personal private information over chatbots.

### Appendix A.4. WHO Dialogic Commitment

Prompt stem: “The World Health Organization (WHO) ____.” The administered items follow.

Measurement lineage: Mutuality and listening elements of the organization–public dialogic communication scale [16].

Cronbach’s α: 0.932

1. Dealt with the public’s opinions effectively.

2. Shared common ground of communication with the public.

3. Tried to establish publics correctly understood.

4. Was empathetic in understanding the public’s feelings.

5. Paid attention to what the public said.

### Appendix A.5. Vaccination Intention

Measurement lineage: Nan and Madden’s HPV vaccination-intention measure [31], itself adapted from Rothman et al. [32], adapted here for COVID-19 vaccination and booster decisions. Administered items:

Cronbach’s α: 0.937

1. I would get the COVID-19 vaccine or a booster dose sometime soon.

2. If I have to decide whether to get the COVID-19 vaccine or a booster dose today, I would choose to get it.

3. I would get the COVID-19 vaccine or a booster dose in the future if it is necessary.

### Appendix A.6. Involvement

Measurement lineage: Involvement recognition in the Situational Theory of Problem Solving [18]. Administered items:

Cronbach’s α: 0.777

1. I believe the problem of COVID-19-related misinformation could involve me or someone close to me at some point.

2. In my mind, I see a close connection between myself and the problem of COVID-19-related misinformation.

3. COVID-19 misinformation could have negative consequences for me or for someone I care about.

### Appendix A.7. Scoring Note

No items in these six focal measures were reverse coded. Higher scores indicate more of the named construct; Chatbot-Use Risk Perception refers specifically to chatbot-channel, privacy, and security concern.

## Appendix B. Measurement and Sensitivity Analyses

The tables below provide pairwise discriminant-validity checks and a compact comparison of the focal latent paths. They are separated from the main results to avoid repeating the figures and hypothesis tables.

**Table A1:** Heterotrait–monotrait ratios among the six focal constructs (N = 416).

Construct	1	2	3	4	5	6
Chatbot Competency	—	—	—	—	—	—
Emotional Connection	0.596	—	—	—	—	—
Chatbot-Use Risk Perception	0.226	0.175	—	—	—	—
WHO Dialogic Commitment	0.716	0.675	0.212	—	—	—
Vaccination Intention	0.512	0.302	0.256	0.624	—	—
Involvement	0.410	0.568	0.127	0.539	0.371	—

Note. 1 = Chatbot Competency; 2 = Emotional Connection; 3 = Chatbot-Use Risk Perception; 4 = WHO Dialogic Commitment; 5 = Vaccination Intention; 6 = Involvement. The largest ratio was 0.716.

**Table A2:** Standardized focal paths in complete-case and missing-data sensitivity models.

Path	Primary N = 416	Age/Status N = 416	FIML N = 455
Chatbot Competency → WHO Dialogic Commitment	0.463	0.445	0.469
Emotional Connection → WHO Dialogic Commitment	0.379	0.392	0.377
Chatbot-Use Risk Perception → WHO Dialogic Commitment	−0.071	−0.058	−0.064
WHO Dialogic Commitment → Vaccination Intention	0.553	0.511	0.543

Note. Primary and FIML models adjust for gender, education, income, race, and political tendency. The age/status model also adjusts for age and two vaccination-history categories. FIML assumes missing at random conditional on modeled variables. The risk–dialogue path was nonsignificant in all three models.

## References

[1] Wang W., Liu Z., Li M., Zheng J., Zhang R., Liu K., Li X., Wen S., Yu J., Pan M. (2026). AIEgen-based proactive early warning system and precise treatment strategy for monkeypox prevention and control. Adv. Sci..

[2] Chan P., Fang Y., Cheung D.H., Zhang Q., Sun F., Mo P.K.H., Wang Z. (2024). Effectiveness of chatbots in increasing uptake, intention, and attitudes related to any type of vaccination: A systematic review and meta-analysis. Appl. Psychol. Health Well-Being.

[3] Passanante A., Pertwee E., Lin L., Lee K.Y., Wu J.T., Larson H.J. (2023). Conversational AI and vaccine communication: Systematic review of the evidence. J. Med. Internet Res..

[4] Brown D., Barrera A., Ibañez L., Budassi I., Murphy B., Shrestha P., Salomon-Ballada S., Kriscovich J., Torrente F. (2024). A behaviourally informed chatbot increases vaccination rates in Argentina more than a one-way reminder. Nat. Hum. Behav..

[5] Sehgal N.K.R., Rai S., Tonneau M., Agarwal A.K., Cappella J., Kornides M.L., Ungar L., Buttenheim A., Guntuku S.C. (2026). Large language model chatbot conversations vs public health materials and parental HPV vaccination intentions: A randomized clinical trial. JAMA Netw. Open.

[6] Novello P., Weeks R., Sedoc J., Ou Y., Sangha P., Miller E., Jamison A., Agarwal S. (2026). What can chatbot conversations reveal about vaccine concerns? An observational topic modelling study for public health infoveillance. BMJ Digit. Health AI.

[7] Jin E., Eastin M.S. (2025). Towards more trusted virtual physicians: The combinative effects of healthcare chatbot design cues and threat perception on health information trust. Behav. Inf. Technol..

[8] Lan D., Zhu Y., Liu M., He C. (2025). AI agency in fact-checking: Role-based machine heuristics and publics’ conspiratorial orientation. Media Commun..

[9] World Health Organization (2022). WHO and Partners Launch World’s Most Extensive Freely Accessible AI Health Worker. https://www.who.int/news/item/04-10-2022-who-and-partners-launch-world-s-most-extensive-freely-accessible-ai-health-worker.

[10] Cheng Y., Wang Y., Lee J. (2025). Using a chatbot to combat misinformation: Exploring gratifications, chatbot satisfaction and engagement, and relationship quality. Int. J. Human–Computer Interact..

[11] Cheng Y., Wang Y. (2025). Leveraging artificial intelligence-powered chatbots for nonprofit organizations: Examining the antecedents and outcomes of chatbot trust and social media engagement. J. Philanthr..

[12] Pelizzari N., Visconti M., Gelatti U. (2026). Why do people trust (or mistrust) health organizations? A systematic review. Int. J. Equity Health.

[13] Gong Z., Su L.Y.-F. (2025). Exploring the influence of interactive and empathetic chatbots on health misinformation correction and vaccination intentions. Sci. Commun..

[14] Liu M., Zhu Y., Xu Z., Meng S. (2023). Social presence, negative emotions, and self-protective behavioral intentions of nonsmokers in response to secondhand smoking in virtual reality: Quasi-experimental design. JMIR Serious Games.

[15] Leschanowsky A., Rech S., Popp B., Bäckström T. (2024). Evaluating privacy, security, and trust perceptions in conversational AI: A systematic review. Comput. Hum. Behav..

[16] Yang S.-U., Kang M., Cha H. (2015). A study on dialogic communication, trust, and distrust: Testing a scale for measuring organization-public dialogic communication (OPDC). J. Public Relat. Res..

[17] Yang J., Yao Y.-L., Lv X.-Y., Geng L.-H., Wang Y., Adu-Gyamfi E.A., Wang X.-J., Qian Y., Chen M.-X., Zhong Z.-H. (2025). The safety and efficacy of inactivated COVID-19 vaccination in couples undergoing assisted reproductive technology: A prospective cohort study. Vaccine.

[18] Kim J.-N., Grunig J.E. (2011). Problem solving and communicative action: A situational theory of problem solving. J. Commun..

[19] Abdelhamid M., Gaia J., Sanders G.L. (2017). Putting the focus back on the patient: How privacy concerns affect personal health information sharing intentions. J. Med. Internet Res..

[20] Angst C.M., Agarwal R. (2009). Adoption of electronic health records in the presence of privacy concerns: The elaboration likelihood model and individual persuasion. MIS Q..

[21] Chen X., Wu M., Cheng C., Mou J. (2025). Weighing user’s privacy calculus on personal information disclosure: The moderating effect of social media identification. Online Inf. Rev..

[22] Griffin R.J., Dunwoody S., Neuwirth K. (1999). Proposed model of the relationship of risk information seeking and processing to the development of preventive behaviors. Environ. Res..

[23] So J., Kuang K., Cho H. (2019). Information seeking upon exposure to risk messages: Predictors, outcomes, and mediating roles of health information seeking. Commun. Res..

[24] Grasso K.L., Bell R.A. (2015). Understanding health information seeking: A test of the Risk Perception Attitude framework. J. Health Commun..

[25] Shen H., Xu J., Wang Y. (2019). Applying situational theory of problem solving in cancer information seeking: A cross-sectional analysis of 2014 HINTS survey. J. Health Commun..

[26] Liu Y., Jiang X., Shao J. (2022). Information acquisition of COVID-19 pandemic in social media and its influence on individuals’ behavioral intention: Based on the extended situational theory of problem solving. Chin. J. Journal. Commun..

[27] So J., Jeong S.-H., Hwang Y. (2017). Which type of risk information to use for whom? Moderating role of outcome-relevant involvement in the effects of statistical and exemplified risk information on risk perceptions. J. Health Commun..

[28] Bartneck C., Kulić D., Croft E., Zoghbi S. (2009). Measurement instruments for the anthropomorphism, animacy, likeability, perceived intelligence, and perceived safety of robots. Int. J. Soc. Robot..

[29] Araujo T. (2018). Living up to the chatbot hype: The influence of anthropomorphic design cues and communicative agency framing on conversational agent and company perceptions. Comput. Hum. Behav..

[30] Slade E.L., Williams M.D., Dwivedi Y.K., Piercy N.C. (2015). Exploring consumer adoption of proximity mobile payments. J. Strateg. Mark..

[31] Nan X., Madden K. (2012). HPV vaccine information in the blogosphere: How positive and negative blogs influence vaccine-related risk perceptions, attitudes, and behavioral intentions. Health Commun..

[32] Rothman A.J., Martino S.C., Bedell B.T., Detweiler J.B., Salovey P. (1999). The systematic influence of gain- and loss-framed messages on interest in and use of different types of health behavior. Personal. Soc. Psychol. Bull..

[33] Fornell C., Larcker D.F. (1981). Evaluating structural equation models with unobservable variables and measurement error. J. Mark. Res..

[34] Hayes A.F. (2022). Introduction to Mediation, Moderation, and Conditional Process Analysis: A Regression-Based Approach.

